# Evolution of Anchor Polymer Systems Used in Arthroscopic Shoulder Surgery—A Comprehensive Review

**DOI:** 10.3390/bioengineering12111146

**Published:** 2025-10-23

**Authors:** Eun-Ji Yoon, Kyeong-Eon Kwon, Jong-Ho Kim

**Affiliations:** Department of Orthopaedic Surgery, Yeouido St. Mary’s Hospital, The Catholic University of Korea, Seoul 07345, Republic of Korea

**Keywords:** shoulder, suture anchors, bioabsorbable polymers, biocomposites

## Abstract

Arthroscopic shoulder surgery has undergone significant evolution over the past decades, particularly in the materials used for suture anchors. The transition from metallic to bioabsorbable polymer anchors has revolutionized soft tissue-to-bone repair procedures, offering distinct advantages in terms of biocompatibility, imaging compatibility, and reduced complications. This comprehensive review examines the current state-of-the-art in anchor polymers used in arthroscopic shoulder surgery and their biocomposite formulations. Additionally, we explore the role of biostable polymers and emerging technologies in anchor design. The review synthesizes clinical outcomes, degradation kinetics, biocompatibility profiles, and mechanical properties of various anchor polymer systems. We also discuss the challenges associated with each material type, including osteolysis, cyst formation, premature degradation, and osseointegration. Recent advances in biocomposite anchors demonstrate promising solutions to address these limitations, offering controlled degradation rates and enhanced osteoconductivity. This review provides clinicians and researchers with a comprehensive understanding of anchor polymer technologies, their clinical applications, and future directions in arthroscopic shoulder surgery. Nevertheless, potential publication bias and heterogeneity among studies should be considered when interpreting comparative data.

## 1. Introduction

Arthroscopic shoulder surgery represents one of the most significant advances in orthopedic surgery, enabling minimally invasive repair of complex soft tissue injuries [1,2,3]. The use of suture anchors has revolutionized orthopedic surgery because it allows for simple and efficient fixation of soft tissue (e.g., tendons and ligaments) to the bone in both open and arthroscopic surgery around the shoulder, elbow, wrist, and lower limb joints [4,5,6]. Shoulder surgery particularly has experienced a significant change in the type of techniques used from open repair of the rotator cuff and labrum using screws, washers, transosseous sutures, and staples to arthroscopic repair using suture anchors [7,8,9].

The evolution of suture anchor materials has been driven by the need to optimize several key factors: mechanical stability, biocompatibility, imaging compatibility, and the ability to facilitate native tissue healing [10,11,12]. Bioabsorbable suture anchors have largely replaced metallic anchors because of concerns of implant loosening, migration, and chondral injury [6,9,13]. Moreover, even metallic anchors have been shown to demonstrate a higher risk of pullout compared with all-suture designs in short-term clinical studies [14]. These limitations have stimulated the development of next-generation polymer-based anchor system to achieve stable fixation while minimizing hardware-related complications [10].

**Figure 1:**
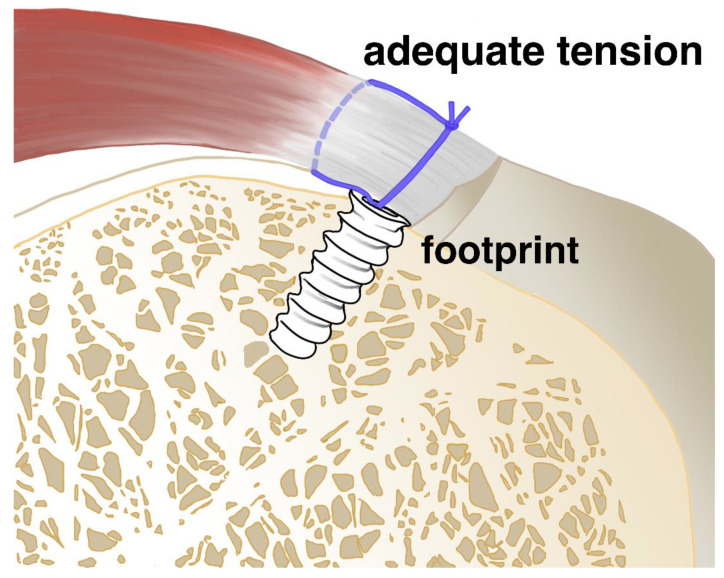
Schematic illustration showing adequate tension and anatomic footprint restoration for tendon-to-bone healing and prevention of loosening or retear.

The primary function of a suture anchor is to maintain secure fixation of soft tissue to bone during the critical healing period, typically 12–24 weeks for most shoulder repairs. The suture anchor serves to attach the tissue at the proper site and maintain its position without loosening or excessive tension until physiologic healing is accomplished, as shown in Figure 1. An ideal anchor should provide adequate initial fixation strength, maintain this strength throughout the healing period, and either integrate permanently with bone or degrade safely without adverse tissue reactions [9,10].

Recent advancements in polymer-based suture anchors include the introduction of biocomposite formulations that incorporate osteoconductive ceramics (β-TCP, calcium sulfate) and the surface modification of PEEK to enhance osseointegration. These materials provide tunable degradation profiles, reduced imaging artifacts, and improved biological integration compared with earlier designs. Emerging nano-engineered polymer composites—such as PLGA or PLLA matrices reinforced with carbon nanotubes, graphene oxide, or nanohydroxyapatite—offer further gains in mechanical strength and osteoconductivity relative to classical polymers [15,16,17,18]. However, challenges remain in balancing degradation rate, maintaining fixation in osteoporotic bone, and preventing cyst formation or foreign body reactions.

This review provides a comprehensive analysis of anchor polymers currently used in arthroscopic shoulder surgery, examining their chemical composition, mechanical properties, degradation kinetics, clinical performance, and associated complications. We also explore emerging biocomposite technologies and future directions in anchor polymer development.

This article is structured as a narrative review, aiming to (1) summarize the evolution and chemical characteristics of anchor polymers, (2) compare their mechanical and biological performance, and (3) provide clinically relevant guidance for material selection based on the latest evidence. Unlike previous systematic reviews that primarily focused on clinical or surgical outcomes, this review emphasizes the integration of polymer chemistry, degradation kinetics, and clinical performance, providing a multidisciplinary perspective that bridges materials science and orthopedic surgery.

## 2. Literature Search Strategy

To ensure comprehensive coverage of both historical and contemporary developments, a structured literature search was conducted in PubMed, Scopus, and Web of Science databases from 1990 to 2025. The search keywords included “suture anchor”, “bioabsorbable polymer”, “PLGA”, “PLLA”, “biocomposite”, “PEEK”, and “rotator cuff repair”. Studies were included if they reported on anchor material composition, degradation kinetics, biomechanical performance, or clinical outcomes. Exclusion criteria comprised non-English language articles, case reports, and animal-only studies. Reference lists of major reviews and relevant clinical studies were manually screened to capture additional sources not retrieved in the database search.

## 3. Historical Evolution of Anchor Materials

### 3.1. From Metallic to Bioabsorbable Anchors

With major advances in arthroscopy, suture anchors became the primary devices used to assist in fixing soft tissues to bone. Metallic anchors were first produced and used in soft tissue fixation around the shoulder [6,19,20]. While metallic anchors, primarily constructed from titanium alloys and stainless steel, provided excellent mechanical properties and proved highly successful in many clinical applications [20], they were associated with several significant drawbacks [21].

The limitations of metallic anchors included:

- Risk of migration into the joint space, potentially causing articular cartilage damage
- Interference with postoperative magnetic resonance imaging (MRI) due to metallic artifacts
- Permanent presence requiring removal during revision procedures
- Stress shielding effects leading to bone remodeling

In addition, their use resulted in some reported complications, including articular surface damage from migrating implants and distortion and artifact production in postoperative magnetic resonance imaging. These complications drove the development of bioabsorbable alternatives that could provide equivalent mechanical fixation while addressing the shortcomings of metallic systems [21].

### 3.2. Introduction of Bioabsorbable Polymers

Bioabsorbable anchors were developed to avoid these problems [22]. Their newer versions demonstrated comparable pull-out strength and similar clinical outcomes to metallic anchors, with a reported lower complication rate [20,23]. The transition to bioabsorbable materials represented a paradigm shift in anchor design field, prioritizing biological integration over permanent mechanical fixation [24,25].

The evolution of bioabsorbable anchors has progressed through several generations:

1. First Generation: Pure polyglycolic acid (PGA) anchors
2. Second Generation: Poly-L-lactic acid (PLLA) anchors
3. Third Generation: Copolymer systems (PLGA, PLDLA)
4. Fourth Generation: Biocomposite anchors with ceramic fillers
5. Fifth Generation: Advanced biocomposites with controlled degradation profiles

## 4. Bioabsorbable Polymer Systems

### 4.1. Polyglycolic Acid (PGA)

Polyglycolic acid (PGA) was among the first bioabsorbable polymers investigated for biomedical use and has been clinically applied as an absorbable suture material since the early 1970s. It was later introduced for suture anchor fabrication, offering the theoretical advantage of complete resorption and replacement by native bone tissue [22,26,27].

#### 4.1.1. Chemical Properties and Degradation

PGA is a linear aliphatic polyester with the chemical formula (C_2_H_2_O_2_)_n_. Its degradation occurs through hydrolytic cleavage of ester bonds, producing glycolic acid as the primary degradation product [28]. The degradation process is characterized by:

- Initial degradation: Begins within the first week after implantation
- Complete resorption: Typically occurs within 6–12 weeks
- Degradation products: Glycolic acid, which is metabolized to carbon dioxide and water

#### 4.1.2. Clinical Limitations

While it was initially also used as a biodegradable anchor, the rapid degradation of PGA and loss of strength shortly after surgery resulted in its discontinuation. Degradation of PGA starts during the first week after anchor implantation; as the glycolic acid products are released, they can cause an inflammatory reaction with synovitis, bursitis, or lytic bone changes [22,26].

The rapid degradation of PGA anchors proved problematic for shoulder repair applications, where tissue healing typically requires 12–24 weeks [29]. The loss of mechanical integrity before adequate soft tissue healing led to poor clinical outcomes and discontinuation of pure PGA anchors [10,30].

### 4.2. Poly-L-Lactic Acid (PLLA)

To address the limitations of PGA, poly-L-lactic acid (PLLA) was developed as an alternative bioabsorbable material with significantly slower degradation kinetics. Later, anchors started to be manufactured using poly-L-lactic acid (PLLA) [24].

#### 4.2.1. Chemical Properties and Degradation

PLLA is a semi-crystalline polymer with higher molecular weight and greater hydrophobicity compared to PGA. Its degradation characteristics include:

- Degradation timeline: 2–5 years for complete resorption
- Degradation mechanism: Hydrolytic cleavage producing lactic acid
- Crystallinity: Higher crystalline content provides greater mechanical strength

It has been shown to degrade very slowly, with complete resorption taking approximately 2–5 years, depending on crystallinity and molecular weight [27,31]. Based on this attribute, poly-lactic acid (PLA), particularly its PLLA form, is not as problematic as PGA; however, very long degradation rates would not allow for complete bony replacement and may create intraosseous foreign body reactions [32].

#### 4.2.2. Clinical Performance

PLLA anchors demonstrated improved clinical performance compared to PGA, with several advantages:

- Maintained mechanical strength throughout the critical healing period
- Reduced inflammatory reactions compared to PGA
- Good biocompatibility profile

However, the extremely slow degradation rate of PLLA created new challenges:

- Prolonged visibility on imaging studies (up to 7 years)
- Risk of foreign body reactions
- Potential for cyst formation around anchor sites

Recent reviews reaffirm that copolymer composition, crystallinity, and molecular weight remain the dominant levers for tuning hydrolytic kinetics in PLA/PLGA systems, with higher glycolide content accelerating water uptake and chain scission, and higher lactide content and crystallinity prolonging strength retention. Updated analyses also emphasize that processing history, such as thermal treatment or polymer orientation, can shift the onset of mass loss and maintain fixation windows relevant to tendon-to-bone healing [31,33,34].

### 4.3. Poly-Lactic-co-Glycolic Acid (PLGA)

To regulate the degradation period of bioabsorbable anchors and reinforce their mechanical properties, copolymers such as poly (D, L-lactide) or PLLA with PGA have been developed. PLGA copolymers represent a significant advancement in bioabsorbable anchor technology, offering tunable degradation rates through manipulation of the lactide-to-glycolide ratio [27,35].

#### 4.3.1. Chemical Composition and Tunable Properties

PLGA copolymers consist of randomly distributed lactic and glycolic acid units, with the ratio determining degradation kinetics:

- PLGA 85:15 (85% lactide, 15% glycolide): Slower degradation (~24 months)
- PLGA 75:25: Intermediate degradation (~18 months)
- PLGA 50:50: Fastest degradation among PLGA formulations (~12 months)

#### 4.3.2. Degradation Kinetics and Clinical Benefits

PLGA showed a resorption time of 24 months. The controlled degradation profile of PLGA offers several clinical advantages:

- Adequate mechanical support during critical healing periods
- Predictable resorption timeline
- Reduced risk of long-term foreign body reactions
- Compatibility with advanced imaging techniques

### 4.4. Transition Toward Metallic and Hybrid Systems

While PLGA copolymers provided a major step toward tunable degradation kinetics, their polymeric nature still limited immediate bone integration and mechanical load-bearing capacity. Recent research has therefore expanded to biodegradable metallic alloys, particularly magnesium-based systems, aiming to combine mechanical robustness with gradual bioresorption. Hsu et al. demonstrated in an in vivo goat shoulder model that a magnesium-alloy (ZK60) suture anchor with MgF_2_ coating maintained fixation strength, promoted osseointegration, and underwent controlled degradation without inflammatory reactions [36]. These findings highlight the potential of biodegradable metallic anchors as a bridge between polymeric systems and next-generation biocomposite materials integrating ceramics or metallic phases for enhanced osteoconductivity.

## 5. Biocomposite Anchor Systems

### 5.1. PLGA/β-Tricalcium Phosphate (β-TCP) Composites

Biocomposite suture anchors are composed of both a biodegradable polymer material and a bone formation-promoting bioceramic material [35,37]. The most commonly used bioceramic is beta-tricalcium phosphate (β-TCP); others include hydroxyapatite, calcium sulfate, and calcium carbonate [10].

#### 5.1.1. Composition and Rationale

Biocomposite anchors typically consist of:

- 70–85% PLGA: Provides mechanical integrity and controlled degradation
- 15–30% β-TCP: Enhances osteoconductivity and bone ingrowth

Poly-lactic co-glycolide (PLGA)/β-TCP is a biocomposite material explicitly developed to promote absorption at a controlled rate. PLGA/β-TCP minimizes inflammatory reaction while promoting osteoconductivity at the implant site via the homogeneously disseminated β-TCP within the absorbable copolymer [38,39].

#### 5.1.2. Clinical Performance

This material has the highest reported osteoconductivity rate. According to literature, almost of suture anchors composed of PLGA/β-TCP were absorbed within 3 years and promoted osteoconductivity with few reported adverse events [10,40].

The superior performance of PLGA/β-TCP composites stems from the synergistic effects of the polymer matrix and ceramic filler:

- β-TCP provides osteoconductive scaffolding for bone ingrowth
- PLGA matrix maintains structural integrity during degradation
- Controlled release of calcium and phosphate ions promotes bone formation

Contemporary biocomposite concepts increasingly leverage nano-scale calcium phosphate to buffer local acidity and present osteoconductive surfaces that encourage earlier bone apposition compared with polymer-only systems [17]. These findings align with reports that triphasic PLGA/β-TCP/CS constructs support staged porosity formation and bone ingrowth while maintaining clinically adequate early fixation [38,41].

### 5.2. Advanced Triple-Component Biocomposites

#### 5.2.1. PLGA/β-TCP/Calcium Sulfate (CS) Systems

Recent biocomposite suture anchor materials are typically composed of PLGA, β-TCP, and calcium sulfate (CS). This triple-component system represents the latest advancement in biocomposite anchor technology, designed to optimize both mechanical properties and biological performance [41].

#### 5.2.2. Degradation Timeline and Benefits

PLGA showed a resorption time of approximately 24 months, depending on the lactide-to-glycolide ratio and implant composition [27,38]. And the other two components have shorter resorption times as shown in animal models (β-TCP, 18 months and CS, 4–12 weeks) [38].

The staggered degradation profile provides several advantages:

1. Early Phase (0–12 weeks): CS degradation creates porosity for cellular infiltration
2. Intermediate Phase (12–18 months): β-TCP provides osteoconductive framework
3. Late Phase (18–24 months): PLGA matrix maintains structural support

The use of this type of suture anchor is associated with: (1) strong primary stability, (2) good healing of the soft tissue, and (3) nearly complete absorption within 24 months [41].

#### 5.2.3. Clinical Evidence

Vonhoegen et al. analyzed 82 PLGA/β-TCP/CS anchors in 48 patients who had undergone arthroscopic rotator cuff repair. They reported that the degradation process seemed to be completed within 21 months, and there was no severe osteolysis or cyst formation observed around any of the 82 anchors. Only two retears occurred, and no anchor pull-out complications were detected [41].

## 6. Biostable Polymer Systems: PEEK

### 6.1. Introduction to PEEK Anchors

Because some biodegradable anchors can be absorbed too quickly, the development of biostable anchors was pursued. Such a biostable anchor—a polyetheretherketone (PEEK) polymer—is synthesized by step-growth polymerization of aromatic dihalides with bisphenol salts, forming a repeating unit of –C_6_H_4_–O–C_6_H_4_–O–C_6_H_4_–CO–(C_19_H_12_O_3_) [42].

#### 6.1.1. Material Properties

PEEK (polyetheretherketone) is a high-performance thermoplastic polymer with exceptional properties for orthopedic applications:

- Chemical resistance: Excellent resistance to hydrolysis and chemical degradation
- Mechanical properties: High strength-to-weight ratio with optimal flexibility
- Biocompatibility: Excellent tissue tolerance with minimal inflammatory response
- Imaging compatibility: Radiolucent properties allowing clear postoperative imaging

PEEK materials showed high strength, strong mechanical properties, good wear- and heat-resistance, and excellent chemical and biological resistance. Therefore, it has many other applications in engineering and medicine [42,43].

#### 6.1.2. Clinical Advantages

PEEK also offers advantages, such as good postoperative imaging and stable fixation, and no complications associated with polymer degradation [5,44,45,46].

The primary advantages of PEEK anchors include:

- Permanent mechanical fixation without degradation
- Superior imaging compatibility for postoperative monitoring
- Excellent biocompatibility with minimal tissue reaction
- Reliable mechanical properties throughout implant lifetime

### 6.2. Limitations and Challenges

Importantly, the major problem with PEEK has been shown to be poor osseointegration. The biologically inert nature of PEEK, while preventing degradation-related complications, also limits its ability to integrate with surrounding bone tissue [43,47].

#### 6.2.1. Osseointegration Challenges

The poor osseointegration of PEEK anchors results from:

- Chemical inertness preventing cellular attachment
- Smooth surface characteristics limiting mechanical interlocking
- Lack of bioactive surface properties

Emerging surface-activation strategies, such as low-temperature plasma modification and bioactive ceramic coatings, have been shown to mitigate PEEK’s limited osseointegration by enhancing osteoblast adhesion and promoting early mineralization, while preserving radiolucency and mechanical stability [48,49,50]. In addition, carbon fiber–reinforced PEEK (CF-PEEK) anchors have shown improved bone ingrowth and radiologic stability compared with conventional PEEK, suggesting enhanced bioactivity and integration potential [51].

#### 6.2.2. Clinical Outcomes and Comparisons

Shoulder function was improved after rotator cuff repair and similar clinical outcomes were achieved regardless of suture anchor material and shape. However, the open-construct PEEK anchor provided better bone ingrowth into the anchor than the non-vented biocomposite anchor at 6 months after arthroscopic rotator cuff repair [5,52]. Similarly, Lee et al. reported that combined use of biocomposite and PEEK anchors during arthroscopic rotator cuff repair achieved comparable functional outcomes and radiologic findings, supporting the clinical interchangeability of these materials [53]. Comparative biomechanical evaluations have shown that PLGA/β-TCP anchors exhibit pull-out strengths comparable to metallic anchors and superior fatigue resistance compared with early-generation PLLA designs [30]. Such findings underline the mechanical viability of biocomposites for high-demand shoulder repairs.

In concert, these results indicate that both PEEK and biocomposite anchors provide reliable fixation and comparable healing, with material selection guided primarily by bone quality, tissue condition, and surgeon preference.

## 7. Clinical Performance and Complications

### 7.1. Bioabsorbable Anchor Complications

#### 7.1.1. Early Complications

Biodegradable suture anchors are also associated with challenges, including problems in the intraoperative or early postoperative period such as (1) implant breakage during anchor insertion, (2) initial fixation loss, (3) incomplete burial of anchors within a bone, which could damage the articular cartilage, and (4) possible anchor migration [6,10,22,39,54].

#### 7.1.2. Osteolysis and Cyst Formation

Screw breakage has been reported in the literature. Glueck et al. reported the case of a 20 year old American football player with osteolysis around the site of insertion of PLLA bioabsorbable suture anchors after 8 months of postoperative follow-up [55]. In a comparative study, Cole et al. observed no significant difference in cyst incidence between all-suture and conventional solid anchors, suggesting that material degradation rather than anchor design plays a dominant role in cyst formation [56]. Recent meta-analyses have shown that perianchor cyst formation remains a frequent finding among biodegradable anchor systems, with reported incidence rates ranging from 10% to 40% depending on polymer composition and surgical technique. These cysts occur more often in bioabsorbable than in non-biodegradable anchors, although most cases are clinically silent and rarely affect functional outcomes [35,54,57]. Ranson et al. further demonstrated that cyst development is multifactorial, resulting from both biochemical degradation effects such as local acidity and polymer by-products, and biomechanical stresses related to anchor insertion angle and cyclic loading, underscoring the combined chemical and mechanical origins of this complication [58]. A more recent systematic review and meta-analysis found that cyst formation around biocomposite anchors most commonly appears within 3 to 12 months postoperatively and is associated with risk factors such as advanced age, large tear size, and medial-row placement of PEEK anchors [59]. This complication remains an important clinical consideration, and Table 1 summarizes its incidence and associated adverse events among different anchor materials.

#### 7.1.3. Loose Body Formation

Biodegradable suture anchors have facilitated and revolutionized arthroscopic tissue-to-bone repair, especially in the shoulder. However, the anchor is but a part of the repair construct, which also includes a suture, tied in a knot, that attaches the tissue (tendon or labrum) to bone. Bioabsorbable anchors may result in loose bodies. The formation of loose bodies represents a unique complication of bioabsorbable anchors, particularly when rapid degradation occurs before adequate tissue healing [60,61].

### 7.2. PEEK Anchor Complications

#### 7.2.1. Perianchor Cyst Formation

While PEEK anchors avoid degradation-related complications, they are still associated with cyst formation [62,63]:

- Lower overall incidence compared to bioabsorbable anchors
- Different mechanism caused by mechanical micromotion rather than material degradation
- Generally smaller and less symptomatic cysts

#### 7.2.2. Revision Surgery Challenges

Revision Bankart repair using PEEK anchors of the same diameter in a pre-existing bone socket is possible but bears high risk of premature anchor failure and can jeopardize the reconstruction [64].

## 8. Degradation Kinetics and Tissue Response

The degradation of bioabsorbable anchors occurs through a well-defined hydrolytic sequence beginning with water uptake and ester-bond cleavage, followed by a gradual reduction in molecular weight, bulk erosion of the polymer, and eventual substitution of the anchor mass with newly formed bone or fibrous tissue. Water penetrates the polymer matrix and induces hydrolysis of ester bands, generating oligomers and acidic byproducts that reduce local pH and accelerate further chain scission (autocatalysis) [38,65]. As the polymer chains shorten, mechanical strength diminishes and bulk erosion takes over, until the implant is resorbed and replaced by native bone or fibrous tissue [66].

This degradation trajectory is modulated by polymer composition and structure. The lactide:glycolide ratio in PLGA profoundly influences degradation kinetics, with higher glycolide content accelerating hydrolysis while higher lactide content retards it [33]. High crystallinity reduces water uptake and slows degradation, preserving structural integrity longer. Molecular weight also matters: larger polymer chains take more time to break down. In copolymer systems with bioactive fillers (e.g., β-TCP or calcium sulfate), these ceramics buffer acidity and provide osteoconductive sites, thereby modulating degradation and fostering bone ingrowth [38,39,67]. Current syntheses highlight that autocatalytic hydrolysis and the microenvironmental pH are major accelerants of bulk erosion in vivo, reinforcing the value of ceramic fillers as local buffers during early resorption [31,33,68].

Local environmental and mechanical conditions further shape degradation. Lower pH, limited perfusion, or restricted fluid exchange slow mass loss; conversely, cyclic loading and micromotion at the bone-implant interface induce microcracks that enhance fluid ingress and hydrolysis [28,69,70,71]. Models of localized enzymatic degradation at interfaces illustrate how degradation may accelerate at surfaces compared to the interior, particularly in composite systems [72].

Clinically, these dynamics explain observed imaging and histologic patterns. Fast-degrading materials like PGA or high-glycolide PLGA often lose fixation before complete tendon-to-bone healing, predisposing to cysts and osteolysis. Slower degrading PLLA or biocomposite anchors maintain stability longer, support gradual bone replacement, but sometimes incite prolonged foreign body reactions (e.g., mild inflammation or granulomas) [10,38,73]. The persistence of biocomposite anchors on MRI beyond two years suggests that complete degradation may lag clinical expectations [10].

Table 2 summarizes the standardized degradation timelines of commonly used anchor materials, highlighting how variations in polymer composition and filler type influence the overall resorption behavior.

## 9. Future Directions and Emerging Technologies

The future of bioabsorbable and polymer-based suture anchors lies in advanced material innovations designed to enhance osseointegration, durability, and biological performance [43]. Polyetheretherketone (PEEK) has favorable mechanical properties but is biologically inert, limiting direct bone integration [42]. To overcome this, various surface modification strategies are being explored. Low-temperature plasma treatment can create nanoscale or microscale surface textures on PEEK, improving osteoblast adhesion [48,77]. Bioactive coatings, such as hydroxyapatite (HA) or other osteoconductive ceramics, are also applied to PEEK to stimulate bone formation and enhance osseointegration [49,78]. Additionally, chemical etching techniques can increase surface roughness, promoting stronger mechanical interlocking between the implant and bone tissue [79].

Beyond PEEK, bioactive polymer surfaces are being developed to actively promote tissue integration [50]. This includes the incorporation of growth factors, such as bone morphogenetic proteins (BMPs), directly into polymer matrices for localized delivery [80]. Surface grafting of bioactive peptides, like RGD sequences, has also been shown to improve cell adhesion and osteogenic differentiation [81]. Furthermore, controlled release systems embedded within polymers allow for sustained release of osteogenic factors, facilitating long-term tissue regeneration [82,83].

The next generation of anchors may utilize stimuli-responsive polymers that adapt to local physiological conditions [84,85]. These advanced materials can be engineered to respond to pH changes, allowing controlled degradation based on the acidic or neutral environment around healing tissue [86,87]. Temperature-responsive polymers can undergo phase transitions to release therapeutic agents when exposed to specific temperature ranges [84]. Similarly, polymers that react to mechanical stress can dynamically alter their stiffness or other properties depending on the biomechanical loads applied [84,86].

Another emerging concept is self-healing polymers, which possess the ability to autonomously repair micro-damage caused by repetitive stress, thereby extending the life of the implant [88,89]. These materials can also adapt to continuous mechanical stress and maintain their structural integrity under cyclic loading, which is especially beneficial for high-demand joints like the shoulder [90].

**Figure 2:**
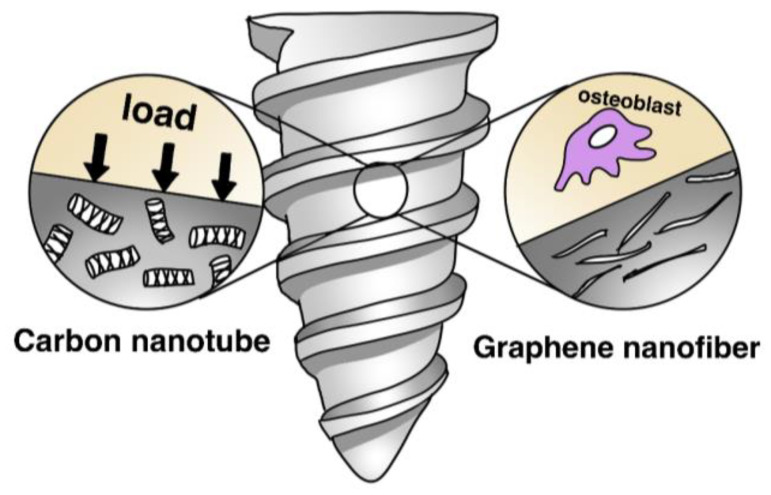
Schematic illustration of a polymeric suture anchor reinforced with carbon nano tubes and graphene nanofibers.

Nanotechnology offers promising pathways for improving anchor performance. Nano-enhanced anchors integrate nanomaterials into polymer matrices to achieve superior functional properties [15]. Carbon nanotubes (CNTs) exceptional mechanical reinforcement, increasing the strength and fatigue resistance of the polymer, while graphene nanofibers increase surface energy to promote osteoblast adhesion (Figure 2) [16]. Recent reviews further highlight that carbon-based nanostructures—including CNTs, graphene nanosheets, and nanofibers—significantly improve load transfer efficiency and interfacial bonding within polymer matrices, resulting in superior fatigue resistance and mechanical stability under cyclic loading conditions [91]. Nanohydroxyapatite (nHA) particles enhance osteoconductivity by mimicking the natural bone mineral structure, encouraging osteoblast proliferation and bone integration [17]. Additionally, Anchors are also being designed with hierarchical structures that combine multiple length scales for optimal performance. At the macro-scale, these anchors maintain strong mechanical stability. The micro-scale porosity allows for cell infiltration and vascularization, while nano-scale surface features improve cellular interactions and signaling, promoting bone ingrowth and integration [18]. This multi-scale approach reflects a biomimetic strategy inspired by natural bone architecture. On the materials side, stimuli-responsive polymers and drug-eluting matrices are poised to tailor local biology, modulating inflammation and osteogenesis during the 12–24-week healing window [84,86]. At the structural level, nano-reinforced composites and biomimetic architectures show promise for enhancing fatigue resistance and bone–implant crosstalk without sacrificing degradability [15,16,17,18].

Future research should now focus on translating these material and structural advances into clinically validated solutions. Integrating advanced manufacturing technologies such as 3D printing and electrospinning can enable customized anchor architectures and controlled degradation profiles [92]. Concurrently, surface-functionalized polymers incorporating osteogenic peptides or localized drug-release systems could improve tendon-to-bone healing while reducing inflammation. Finally, multicenter clinical trials using standardized imaging and biomechanical outcome measures are essential to confirm long-term safety, optimize anchor design, and establish evidence-based guidelines for material selection.

## 10. Clinical Decision-Making Guidelines

The selection of appropriate suture anchor material is a critical factor in optimizing outcomes for shoulder and other orthopedic soft tissue repair surgeries. The decision should be based on a comprehensive assessment of patient factors, surgical considerations, and material properties, ensuring a balance between mechanical stability, biological healing response, and clinical objectives [10].

### 10.1. Patient Factors

Patient-specific variables significantly influence material selection. Age and activity level play a major role; for instance, young or high-demand patients require anchors with greater mechanical strength and slower degradation to withstand higher loads during rehabilitation. Bone quality and density are also important, elderly or osteoporotic patients may necessitate anchors with enhanced fixation strength, such as titanium or PEEK-based designs to mitigate risk of early loosening in low-density bone [10,93]. Patients with a history of prior surgeries may present with altered bone architecture or scar tissue, requiring careful consideration of anchor design and placement. Finally, healing potential is affected by systemic factors such as diabetes, smoking, or autoimmune disease, which can influence the rate of tendon-to-bone integration and may favor the use of bioactive or slower-degrading anchor materials [94,95,96].

### 10.2. Surgical Factors

The complexity and size of the repair dictate the mechanical demands on the anchor. Larger or multi-tendon tears, such as massive rotator cuff tears, require anchors with higher pullout strength and fatigue resistance [96]. Revision surgeries are particularly challenging because of compromised bone stock and previous hardware, often necessitating specialized anchor designs such as all-suture or biocomposite systems [10,96,97]. The expected postoperative mechanical loading is another determinant; for example, early active rehabilitation protocols demand materials with high initial stability [10]. Additionally, surgeon preference and experience influence anchor choice, as familiarity with specific insertion techniques can affect both procedural efficiency and clinical outcomes.

### 10.3. Material Properties

Anchor choice should match the healing timeline and local biology. Fast-degrading polymers (e.g., PGA-rich) risk premature strength loss, whereas very slow-resorbing polymers (e.g., pure PLLA) may persist for years and occasionally provoke foreign body reactions [10,38,41]. Biocomposites (PLGA/β-TCP ± CS) provide a practical balance, offering adequate early strength, buffering of acidity, and osteoconductivity, with near-complete resorption occurring within approximatel 24–36 months [38,39,41]. PEEK provides stable, radiolucent fixation without degradation-related issues and is attractive in osteoporotic bone or revision settings, though osseointegration can be limited [10,52]. Imaging consideration also matter: metallic anchors may cause MRI artifact, whereas polymer/biocomposite/PEEK options facilitate postoperative evaluation [10].

Although biocomposites and PEEK dominate current use, alternative designs such as all-suture and metallic hybrid anchors remain clinically relevant, particularly in cost-sensitive or revision-limited settings [6,54].

### 10.4. Evidence-Based Framework

Table 3 presents a synthesized framework that integrates patient-, surgery-, and material-related considerations into a practical guide for suture-anchor selection. Rather than prescribing a single ideal material, this framework emphasizes the importance of aligning mechanical performance, biological response, and the clinical environment in which healing occurs.

For young and active patients, biocomposite anchors composed of PLGA and β-TCP, with or without calcium sulfate, offer an effective combination of fixation strength, degradation control, and osteoconductivity. In elderly or osteoporotic patients, PEEK anchors are preferred because they maintain stable fixation regardless of bone density and avoid degradation-related complications. Revision procedures also benefit from PEEK due to its radiolucency and non-resorptive nature, allowing secure fixation and clear postoperative imaging.

In large or high-tension rotator-cuff repairs, sustained mechanical stability is paramount, making PEEK or reinforced biocomposites appropriate choices. Conversely, procedures such as Bankart repair, which rely heavily on biological integration, may favor biocomposite anchors for their osteoconductive potential while maintaining adequate fixation strength.

This integrative framework highlights the interplay among patient biology, mechanical demand, and material behavior. Applying an evidence-based decision matrix enables surgeons to select the most appropriate anchor system for each clinical context, thereby optimizing both mechanical reliability and biological healing.

## 11. Conclusions

The evolution of anchor polymers in arthroscopic shoulder surgery represents a remarkable advancement in orthopedic biomaterials. From the early challenges with rapid degradation of PGA anchors to the sophisticated biocomposite systems available today, each generation has addressed specific clinical limitations while introducing new considerations. Current evidence supports the use of biocomposite anchors, particularly PLGA/β-TCP/CS systems, for most primary shoulder repair procedures. These materials offer an optimal balance of mechanical properties, controlled degradation, and enhanced osteoconductivity. Compared to PLLA and PDLDA, PLGA/β-TCP/CS seems to have superior characteristics regarding degradation time and the occurrence of osteolysis and cyst formation.

PEEK anchors remain valuable for specific clinical scenarios, particularly revision surgeries and cases requiring permanent fixation. The development of open-construct PEEK anchors with enhanced venting has addressed some osseointegration concerns, making them increasingly attractive for certain applications.

In parallel, robotic-assisted arthroscopic systems are being developed to enhance anchor placement precision and reproducibility, particularly in complex shoulder reconstructions [98,99]. Complementing these advances, artificial intelligence-assisted navigation and predictive modeling have also been applied to shoulder arthroscopy, enabling automated anchor trajectory planning and postoperative outcome prediction [100,101].

In concert, these emerging technologies represent an important procedural evolution that parallels ongoing material innovation. The future of anchor polymer technology lies in smart materials that can adapt to local tissue conditions, provide controlled drug delivery, and actively promote tissue regeneration. As our understanding of polymer degradation kinetics and tissue healing biology continues to advance, we can expect further refinements in anchor design that will improve clinical outcomes while minimizing complications. Clinicians must remain informed about the properties and performance characteristics of different anchor materials to make optimal choices for individual patients. The continued development of evidence-based guidelines for material selection will be crucial for maximizing the benefits of these advanced polymer systems in arthroscopic shoulder surgery.

## Figures and Tables

**Table 1:** Clinical complications associated with different bioabsorbable suture anchors.

Anchor Type	Cyst Formation Rate	Severe Cyst Rate	Other Reported Complications	Timeline	Reference
PLLA	15–30%	5–10%	Inflammatory reaction, osteolysis, foreign body response	12–24 months	[32,35]
PLGA/β-TCP	40–50%	10–15%	Mild osteolytic changes	6–18 months	[41]
PLGA/β-TCP/CS	<5%	<2%	Minimal osteolysis; no anchor pull-out or migration	12–21 months	[54]

Data show the reported frequency of perianchor cyst formation and other adverse events for each material type. Complication rates were extracted or converted to approximate percentages for cross-comparison between studies.

**Table 2:** Standardized comparison of degradation kinetics among commonly used anchor polymers.

Material	Initial Strength Loss	50% Mass Loss	Complete Resorption	Reference
PGA	2–4 weeks	6–8 weeks	12–16 weeks	[27,28]
PLLA	6–12 months	18–36 months	36–60 months	[27,28,31,74]
PLGA (85:15)	6–12 months	12–18 months	24–30 months	[27,38,39]
PLGA/β-TCP	8–12 months	18–24 months	30–36 months	[38,39,75,76]
PLGA/β-TCP/CS	6–9 months	15–21 months	21–24 months	[41]

Degradation periods were adjusted to comparable timeframes for cross-study consistency.

**Table 3:** Suture-anchor material recommendations by clinical scenarios.

Clinical Scenario	First Choice	Second Choice	Rationale
Primary rotator cuff repair (young patient)	PLGA/β-TCP/CS	PLGA/β-TCP	Optimal degradation profile and osteoconductivity
Primary rotator cuff repair (elderly patient)	PEEK	PLGA/β-TCP	Stable fixation in low-density bone; avoids degradation issues
Revision surgery	PEEK	PLGA/β-TCP	Preserves bone stock and ensures radiolucent fixation
Large/massive tears	PEEK	PLGA/β-TCP	High mechanical strength and fatigue resistance
Bankart repair	PLGA/β-TCP	PEEK	Balanced mechanical and biological performance

Recommendations synthesized from [5,10,38,41,42,54,96].

## Data Availability

No new data were created or analyzed in this study.

## Abbreviations

The following abbreviations are used in this manuscript:

PGA	polyglycolic acid
PLLA	poly-L-lactic acid
PLGA	poly-lactic-co-glycolic acid
β-TCP	beta-tricalcium phosphate
CS	calcium sulfate
PEEK	polyetheretherketone
